# Clinical Factors Influencing the Efficacy of Systemic Moxifloxacin in the Therapy of Patients With Generalized Aggressive Periodontitis: A Multilevel Analysis From a Clinical Trial

**DOI:** 10.5539/gjhs.v8n3p80

**Published:** 2015-06-25

**Authors:** Carlos M. Ardila, Isabel C. Guzmán

**Affiliations:** 1 Biomedical Stomatology Research Group, Department of Periodontology, School of Dentistry, Universidad de Antioquia U de A, Medellín, Colombia

**Keywords:** clinical trial, generalized aggressive periodontitis, moxifloxacin, multilevel analysis

## Abstract

**Background:**

It has been reported that clinical results of mechanical periodontal treatment could differ between subjects and among different sites of the tooth in the patient. The objective of this multilevel analysis is to investigate clinical factors at subject and sites of the tooth that influence variations in clinical attachment (CAL) increase and probing depth (PD) diminution of adjunctive moxifloxacin (MOX) at six months post-treatment in generalized aggressive periodontitis.

**Methods:**

This clinical trial included 40 patients randomly distributed to two therapy protocols: scaling and root planing alone or combined with MOX. Multilevel linear models for continuous variables were formulated to evaluate the clinical impact of the hierarchical configuration of periodontal data.

**Results:**

Six months following therapy, the divergences between both protocols were statistically significant in PD diminution and CAL increase, favouring the MOX therapy (p<0.001). Besides, the multilevel analysis revealed that adjunctive MOX at the subject level, non-molar and the interaction non-molar x MOX at the tooth level, interproximal sites and the interaction interproximal sites x MOX at the site level, were statistically significant factors in determining CAL increase and PD diminution.

**Conclusions:**

The main cause of variability in CAL gain and PD reduction following adjunctive MOX was attributable to the tooth level. Adjunctive MOX and their interactions with non-molar and interproximal sites showed higher clinical benefits at the tooth and site levels which could be essential for PD reduction and CAL gain in generalized aggressive periodontitis subjects.

## 1. Introduction

Aggressive periodontitis shows a rapid attachment loss related to compromised host immune reaction and greatly pathogenic microorganisms (Teughels, Dhondt, Dekeyser, & Quirynen, 2014). A recent systematic review and meta-analysis indicated that for the management of aggressive periodontitis subjects, adjunctive antibiotics plus mechanical therapy occasioned a considerable supplementary benefit than mechanical treatment alone; a tendency presented that amoxicillin plus metronidazole (AMOX+METRO) is the most effective antimicrobial protocol (Keestra, Grosjean, Coucke, Quirynen, & Teughels, 2014). Also, a clinical study documented that systemic moxifloxacin (MOX) leads to better advantages than scaling and root planing (SRP) in subjects with aggressive periodontitis (GAgP) (Ardila et al., 2015). Adjunctive AMOX+METRO (Keestra et al., 2014; Guerrero et al., 2005) and MOX (Ardila et al., 2015) have showed higher benefits in clinical attachment level (CAL) increase and probing depth (PD) diminution in comparation to SRP alone in aggressive periodontitis.

Nevertheless, it has been reported that clinical results of mechanical therapy could differ not simply among subjects but likewise among different sites of the tooth in the patient (Van der Weijden & Timmerman, 2002). This concern has been elucidated by various researchers in periodontitis patients treated with SRP, adjunctive antibiotics, surgical therapy and combinations of these therapies (D’Aiuto, Ready, Parkar, & Tonetti, 2005; Tomasi, Koutouzis, & Wennström, 2007; Mdala et al., 2012; Kim, Schenk, Lungeanu, Reitmeir, & Eickholz, 2007; Mombelli et al., 2013). Most of these clinical studies have contemplated chronic periodontitis patients, but almost nothing is known in aggressive periodontitis subjects. Also, a meta-analysis (Keestra et al., 2014) indicated that the efficacy of AMOX+MET in aggressive periodontitis subjects is lower after 3 months than the efficacy in chronic periodontitis subjects; nevertheless, the efficacy of AMOX+MET was advanced at six and twelve months in aggressive periodontitis than in chronic periodontitis subjects. Besides, systematic reviews (Herrera et al., 2002; Haffajee et al., 2003) have recommended that the additional advantage estimated from antimicrobial treatment could be better in subjects with aggressive periodontitis. These results could indicate that adjunctive antibiotics have not the same effect in chronic than in aggressive periodontitis patients.

Considering some particularities of aggressive periodontitis, namely, age of beginning, periods of advance, forms of damage and clinical evidence of swelling (Armitage & Cullinan, 2010), among others, the influence of the fundamentally ordered configuration of periodontal figures in the treatment of aggressive periodontitis must be explored. It is coherent to contemplate that based on these particularities periodontal treatment could diverge not simply between individuals but also among tooth sites in aggressive periodontitis patients.

To our understanding, no investigations have assessed the influence of the hierarchical organization of periodontal information in determining the variability of the clinical outcomes after adjunctive MOX in aggressive periodontitis. Also, adjunctive MOX has been scarcely studied in clinical trials, particularly in aggressive periodontitis. Thus, the aim of this multilevel analysis was to investigate clinical factors that influence variations in clinical attachment increase and probing depth diminution of adjunctive MOX compared to SRP alone at six months post-treatment in GAgP patients.

## 2. Method

Comprehensive descriptions of the clinical trial design containing patients’ selection, randomization, allocation, therapy, adherence and clinical evaluation have been previously published (Ardila et al., 2015). A concise description is given here.

### 2.1 Subjects

The subjects had at minimum twenty teeth, excepting third molars and teeth designated for removal. The study protocol was permitted by the Ethics Board on the Faculty of Dentistry of the Universidad de Antioquia agreeing to the Declaration of Helsinki. All individuals were informed independently concerning the purposes, possible dangers and advantages of the therapies and signed the informed agreement.

The diagnosis of GAgP was made following the recommendations of the American Academy of Periodontology (Armitage, 1999).

### 2.2 Investigational Scheme and Therapy

Both therapies included mechanical therapy plus adjunctive MOX (400 mg one per day for seven days) or SRP plus placebo (control group) one per day for seven days. An equalized chance block system was accomplished to organize the randomization sequence to evade unequal equilibrium between both therapies. The randomization list was referred to a clinical director no involved in the investigation, which applied the distribution. Consequently, therapy allocations were distributed to numbered obscure packets.

### 2.3 Clinical Evaluation

Patients were examined at baseline and at six months after treatment. At each checking appointment, detectable plaque, bleeding on probing (BOP), PD and CAL were determined at six sites of each tooth (excepting third molars). The PD and CAL lengths were documented to the closest millimeter by a standardized probe (UNC-15, Hu-Friedy, Chicago, IL).

The same blinded, qualified and standardized clinician completed the evaluation at all appointments for selected patients. The examiner dentist did not execute the treatment on the patients. The intra-examiner concordance was calculated before and through the experimental phase. The intra-class concordance for average CAL and PD were 0.91 and 0.92, correspondingly.

### 2.4 Primary and Secondary Outcome Variables

In the present multilevel analysis, a change in CAL regarding baseline and six months (ΔCAL) was measured as the primary outcome characteristic. Secondary result variable contained changes for the average variations of PD. Consequently, a difference in PD concerning baseline and six months (ΔPD) was considered as a dependent variable.

The sample size to guarantee sufficient power was estimated contemplating changes of one mm for CAL and a standard deviation of 1 mm between treatments (Varela et al., 2011). Considering these estimates, it was established that ≥12 patients per protocol would be required to supply 80% power with an α of 0.05. To compensate drop-out proportion, 20 subjects were enlisted per therapy protocol.

### 2.5 Statistical Analysis

Changes in quantitative and qualitative parameters were assessed by independent t-test (data were distributed normally) and *X*^2^ test, respectively. Independent t-test was executed to define the changes between treatments concerning differences in clinical parameters (CAL and PD). A repeated-measures ANOVA was performed to identify intra-group changes in clinical factors. These analyses were implemented operating a statistical software (Statistical Package for the Social Sciences, version 18, Chicago, IL). The alpha was established to 5%.

Three levels of variability were demarcated: the subject, the tooth, and the site. Patient factors incorporated age (years), gender, plaque and BOP scores and treatments (Adjunctive MOX versus SRP+placebo). Tooth parameters included one categorical variable: molars/non-molars. Site characteristics considered location (interproximal versus buccal/lingual). Variance models (empty models) were created calculating differences in CAL (ΔCAL) and PD (ΔPD) regarding baseline and six months as dependent variables without incorporating explanatory variables. The empty models were computing considering the complete variation of ΔCAL and ΔPD and to assign it to the subject, tooth, and site levels. A sequence of explicatory factors was formerly analysed (multivariate models) in order to calculate the association between each explicatory variable and the dependent variable. When the explanatory variable was statistically significant, the interaction with MOX was also included in the multivariate model.

Consequently, multilevel linear models for continuous variables were analysed, incorporating examinations for the normality of the residuals at the diverse levels. Multicollinearity examination was executed for each independent variable. Regression coefficients were calculated operating iterative general least squares. Nested models were examined for significant advances in model fitting by relating the diminution in -2 log likelihood (-2LL) with a *X*^2^ allocation. All multilevel analyses were completed executing a statistical package (MLwin 2.02, London, UK). The alpha was established to 5%.

## 3. Results

This experimental study had six months of development. A total of 40 subjects who joined the dental clinics of the Universidad de Antioquia, Medellín, Colombia, were enrolled between February 2012 and August 2013.

Of the 40 patients enrolled, 36 patients had all the information for all examinations while four patients had one absent appointment. Intent-to-treat analyses were executed in the 4 patients with absent information; consequently the final examination was passed forward, offering a total of 40 patients with full information that were involved in the analyses. Flow chart of the trial design was explained earlier (Ardila et al., 2015).

Patients enlisted in the clinical trial described complete adherence to the recommended sequence of MOX and placebo and nobody informed unfavorable episode related with the treatment.

The baseline features of the patients have been formerly presented (Ardila et al., 2015); there were no observed dissimilarities between protocols for socio-demographic characteristics.

The factors related with the three levels that were investigated are specified in Table 1.

**Table 1:** Age, gender and baseline parameters associated with patient, tooth and site levels

Parameter	Value
Patients	*N*= 40
Age (mean±SD)	27.4±1
Gender (male/female)	17/23
Treatment (Moxifloxacin/mechanical therapy)	20/20
Plaque score (mean±SD)	44±13
Bleeding on Probing score (mean±SD)	46±13
Tooth	*N*=1079
Site	*N*=6474
Probing Depth (mean±SD)	4.3±0.4
Clinical Attachment Level (mean±SD) Position (db/b/mb/dl/l/ml)	4.9±0.5 1079 each one

SD: standard deviation; db: distobuccal; b: buccal; mb: mesiobuccal; dl: distolingual; l: lingual; ml: mesiolingual.

The data involved 6474 tooth sites at 1079 teeth in 40 patients. Variations in CAL and PD in the two groups through the trial phase are observed in Table 2. In both treatments protocols a significant diminution of PD and CAL increase was observed (p<0.0001), and this change was preserved after six months. The changes between therapies were significant after treatment, favouring the MOX group (p<0.001).

**Table 2:** Changes in the clinical parameters in the two protocols during the trial phase

Parameter	Moxifloxacin		Control group	
	Baseline	6 months	Baseline	6 months
Clinical Atthacment Level (mean±SD)	4.92±0.5*	3.14±0.6**	4.93±0.4*	3.77±0.4**
Probing Depth (mean±SD)	4.27±0.4*	3.08±0.6**	4.34±0.5*	3.5±0.4**

SD: standard deviation;

* Changes were detected among the two time evaluations (repeated-measures ANOVA p<0.001);

** Changes were perceived between the treatments (*t* -test *p*<0.001).

Results from the empty multilevel models with ΔCAL and ΔPD as the dependent variables are presented in Tables 3 and 4.

**Table 3:** Hierarchical regression analysis assessing the influence of subject, tooth and site characteristics in the variability of clinical attachment level (CAL) gain

Intercept	ΔCAL Baseline-6months	

	Empty model β±SE	Multivariate model β±SE
	2.974±0.095	3.743±0.225
Patient (Level 3)	0.317±0.081 (16) ‡	0.237±0.059 (-25%)•
Tooth (Level 2)	1.183±0.054 (60) ‡	0.732±0.034 (-38%)•
Site (Level 1)	0.461±0.009 (24) ‡	0.308±0.006 (-33%)•
Total variance	1.961	1.277
-2 LL	17083.055	12305.077*

‡ Proportion of variability in ΔCAL provided by the multilevel analysis at the subject, tooth, and site levels.

• Change in the proportion of variability in ΔCAL at the subject, tooth, and site levels when explicatory factors were incorporated in the analysis.

* Variation in -2 LL was significant (*P*<0.001).

**Table 4:** Hierarchical regression analysis assessing the influence of subject, tooth and site characteristics in the variability of probing depth (PD) reduction

Intercept	ΔPDBaseline-6months	

	Empty model β±SE	Multivariate model β±SE
	2.816±0.084	3.622±0.264
Patient (Level 3)	0.246±0.063 (17)‡	0.203±0.051 (-17%)†
Tooth (Level 2)	0.885±0.040 (61)‡	0.711±0.033 (-20%)†
Site (Level 1)	0.325±0.006 (22)‡	0.304±0.006 (-9%)†
Total variance	1.456	1.218
-2 LL	14790.139	10193.559*

‡ Proportion of variability in ΔPD provided by the multilevel analysis at the subject, tooth, and site levels.

† Change in the proportion of variability in ΔCAL at the subject, tooth, and site levels when explicatory factors were incorporated in the analysis

* Variation in -2 LL was significant (*P*<0.001)

The empty model for ΔCAL provided a total unexplained variance of 1.96, the majority attributed to variation between teeth (60%), followed by between sites (24%) and between subjects (16%). The addition of the explanatory variables occasioned a 35% diminution of the entirely inexplicable variability: 25%, 38% and 33% at the subject, tooth and site levels, respectively. Appreciably superior fit was reached introducing the clinical explicatory variables at the three levels (P<0.05) (Table 3).

The empty model for ΔPD presented a total unexplained variance of 1.46, the majority accredited to variation between teeth (61%), followed by between sites (22%) and between subjects (17%). The insertion of the explanatory variables conducted to a 17% diminution of the absolutely inexplicable variability: 17%, 20% and 9% at the subject, tooth and site levels, respectively. Meaningfully improved fit was accomplished including the clinical explanatory variables at the three levels (P<0.05) (Table 4).

Table 5 depicts the multilevel multivariate model studying the explicatory variables analysing ΔCAL as the outcome variable. At the subject level, adjunctive MOX showed a significantly more positive response in terms of CAL gain (P=0.02). At the tooth level, the analysis demonstrated that non-molars presented the best significant gains in CAL than molars (P<0.0001). Furthermore, the interaction non-molar x MOX was significantly associated with CAL gain at six months (P<0.0001). Finally, at the site level, interproximal locations were the zones where CAL gains were superior to at the buccal/lingual sites (P=0.02). Also, the interaction interproximal sites x MOX was significantly associated with CAL gain at six months (P<0.0001).

**Table 5:** Hierarchical regression analysis assessing the influence of subject, tooth and site factors, describing the variance in clinical attachment level (CAL) gains

Parameters	ΔCAL Baseline-6months (β±SE)	P value
Subject Level		
Adjunctive MOX/SRP+placebo	0.620±0.309	0.02
Tooth Level		
Tooth position (non-molar/molar)	0.845±0.069	<0.0001
Non-molar x MOX	-0.311±0053	<0.0001
Site Level			
(db-mb-dl-ml/b-l)	0.055±0.028	0.02
(db-mb-dl-ml) x MOX	-0.295±0.026	<0.0001

db: distobuccal; b: buccal; mb: mesiobuccal; dl:distolingual; l: lingual; ml: mesiolingual.

A similar tendency shows the multilevel multivariate model investigating the explanatory variables influencing ΔPD as the dependent variable (Table 6). At the patient level, adjunctive MOX showed a significantly more advantageous response in terms of PD reduction (P=0.04). At the tooth level, the analysis demonstrated that non-molars presented the best reductions in PD than molars (P<0.0001). Moreover, the interaction non-molar x MOX was significantly associated with PD reduction at six months (P<0.0001). Also, at the site level, interproximal positions were the parts where PD reductions were superior to at the buccal/lingual sites (P=0.01). Besides, the interaction interproximal sites x MOX was significantly associated with PD reduction at six months (P=0.02).

**Table 6:** Hierarchical regression analysis assessing the influence of subject, tooth and site factors, describing the variance in probing depth (PD) reduction

Parameters	ΔCAL Baseline-6months (β±SE)	P value
Subject Level		
Adjunctive MOX/SRP+placebo	0.781±0.454	0.04
Tooth Level			
Tooth position (non-molar/molar)	0.817±0.058	<0.0001
Non-molar x MOX	-0.914±0.185	<0.0001
Site Level		
(db-mb-dl-ml/b-l)	0.045±0.020	0.01
(db-mb-dl-ml) x MOX	-0.130±0.064	0.02

db: distobuccal; b: buccal; mb: mesiobuccal; dl:distolingual; l: lingual; ml: mesiolingual.

## 4. Discussion

The application of surrogate parameters such as PD and CAL to estimate the clinical success of several therapies is a usual method (Greenstein, 2005). In this report, variations in CAL and PD between baseline and 6 months after adjunctive MOX to one-stage full-mouth SRP compared to SRP alone were evaluated using a multilevel approach. Consequently, the main cause of variability in CAL gain and PD reduction following adjunctive MOX was referable to the tooth level, followed by the site and the subject levels. Comparable effects were documented in a previous multilevel analysis relating nonsurgical and surgical therapy in subjects with aggressive and chronic periodontitis (Kim et al., 2007). Also, merely elements at the tooth position level were recognized by a multilevel model as significant for the management result of adjunctive doxycycline for the re-instrumentation of pathologic sites in subjects with chronic periodontitis (Tomasi et al., 2008).

In the present research, the final model for ΔCAL, including all of the significant factors conducted to a 35% diminution of the entirely inexplicable variability. Besides, the final model for ΔPD conducted to a 17% decrease of the total inexplicable variation being higher at the tooth level. These conclusions focus the significance of considering factors related with the tooth level in order to elect periodontal therapy properly. In line, the observations reported by Tomasi et al. (2007) recommend focusing on factors at the tooth level in order to explore for supplementary elements that may impact the projection of the result of pathologic pockets in patients with chronic periodontitis. A similar recommendation could apply in aggressive periodontitis.

In the current study, the multilevel models associated CAL gain and PD reduction with adjunctive MOX (at the patient level); non-molar and the interaction non-molar x MOX (at the tooth level); and interproximal sites and the interaction interproximal sites x MOX (at the site level). The significant interactions between MOX with non-molar and interproximal sites show that MOX could be a crucial factor for PD reduction and CAL gain in GAgP.

To the best knowledge of the authors, this is the initial experimental study that evaluates the influence of different factors at the subject, tooth and site levels in the adjunctive MOX treatment of patients diagnosed with GAgP. Previous investigations have studied subjects with chronic and aggressive periodontitis managed with various forms of therapies (D’Aiuto et al., 2005; Tomasi et al., 2007; Mdala et al., 2012; Kim et al., 2007; Mombelli et al., 2013). Curiously, the efficacy of systemic MOX in chronic periodontitis using a multilevel approach has not been evaluated previously. However, the efficacy of adjunctive MOX regarding PD diminution and CAL increase has been also perceived in chronic periodontitis patients (Guentsch et al., 2008). Also, using multilevel models, Mdala et al. (2012) showed that adjunctive AMOX+MET presented more clinical advantages than others therapies. Overall, our results corroborate the published information presented earlier showing that treatment results in dissimilar positions and teeth in the identical subject are not independent (Tomasi et al., 2007; Mdala et al., 2012; Kim et al., 2007).

In this report, worst results were detected in molars, which is in agreement with conclusions informed by others researchers who applied hierarchical approaches in the evaluation of the therapy effect (D’Aiuto et al., 2005; Tomasi et al., 2007; Mdala et al., 2012); this observation may be related to difficult accessibility for subgingival SRP in molars (Tomasi et al., 2007). Kim et al. (2007) in a multilevel analysis reported more pronounced PD diminution and CAL increase in single-rooted teeth. In line, Mombelli et al. (2013) indicated that in non-molar that were treated with AMOX+MET fewer sites remained with PD>4mm and BOP. Also here, the interaction non-molar and MOX was associated with greater PD reduction and CAL gain.

This multilevel analysis showed that at the site level more diminutions in PD were detected for interdental positions than in buccal/lingual sites. This is in conformity with preceding information (D’Aiuto et al. 2005; Tomasi et al., 2007) and coherent with the predominant situation of profounder pockets in the interdental spaces (D’Aiuto et al., 2005). In this report, the interaction interproximal sites and MOX was associated with higher PD diminution and CAL increase. In accordance, a recent meta-analysis indicated that systemic antibiotics showed a significant additional PD diminution and CAL increase for modest and profound pockets (Keestra et al., 2014). Also in this investigation, the use of adjunctive MOX resulted in CAL increase and PD diminution with data comparable with previous studies that used AMOX+METRO in GAgP (Aimetti, Romano, Guzzi, & Carnevale, 2012; Mestnik et al., 2012).

The current report did not observe any influence of age, gender, plaque and BOP at the patient level on CAL gain and PD reduction. Comparable inferences have been informed in multilevel studies (Tomasi et al., 2007; D’Aiuto et al., 2005; Kim et al., 2007; Tomasi et al., 2008). As was reported here, a relatively modest influence of patient's parameters in multilevel analysis has been generally documented (D’Aiuto et al., 2005); D’Aiuto et al. (2005) suggest that patients with significant systemic conditions may cause more variability. In concordance with a previous multilevel studied where mechanical and surgical periodontal treatments were compared in aggressive and chronic periodontitis patients, more factors related with tooth level affecting therapy results than subject related features (Kim et al., 2007).

Although in the present report smokers were excluded, multilevel studies revealed that smoking revealed a adverse influence on the magnitude of PD reduction (Tomasi et al., 2007) and CAL gain (Kim et al., 2007) in patients treated with nonsurgical (Tomasi et al., 2007; Kim et al., 2007) and surgical therapies (Kim et al., 2007).

One weakness of this multilevel analysis is the six-month estimation phase. Categorically, a longer prospective observation of these patients will be indispensable to conclude if this adjunctive treatment would generate determined satisfactory modifications in the periodontal clinical parameters over time.

In conclusion, adjunctive MOX, non-molar and the interaction non-molar x MOX, interproximal sites and the interaction interproximal sites x MOX were factors in determining CAL increase and PD diminution in GAgP. The main cause of variability in CAL increase and PD diminution following adjunctive MOX was attributable to the tooth level. Finally, adjunctive MOX and their interactions showed higher clinical benefits at the tooth and site levels.
